# Real-World Evidence to Reinforce Clinical Trial Evidence in Health Technology Assessment: A Critical Review of Real-World Evidence Requirements from Seven Countries and Recommendations to Improve Acceptance

**DOI:** 10.3390/jmahp12020009

**Published:** 2024-05-20

**Authors:** Katia Thokagevistk, Céline Coppo, Laetitia Rey, Amanda Carelli, Veronica Díez, Sarah Vaselenak, Liana Oliveira, Ajay Patel, Emilia Sicari, Teresa Ramos, Susanne Schach, Erika Schirghuber, Alex Simpson, Remy Choquet, Katell Le Lay

**Affiliations:** 1 Roche, 4 Cr de l’Île Seguin, 92100 Boulogne-Billancourt, France; 2 IQVIA, 17 bis Tsse. des Reflets, 92400 Courbevoie, France; 3 Roche, Rua Dr. Rubens Gomes Bueno, 691—Várzea de Baixo, São Paulo 04730-903, Brazil; 4 F. Hoffmann-La Roche Ltd., C/Ribera del Loira 50, 28042 Madrid, Spain; 5 Roche, 7070 Mississauga Rd, Mississauga, ON L5N 5M8, Canada; 6 Roche Products Ltd., Hexagon Place, Shire Park, Falcon Way, Welwyn Garden City AL7 1TW, UK; 7 Roche SpA, Viale Gian Battista Stucchi, 110, 20900 Monza, Italy; 8 Roche, Emil-Barell-Straße 1, 79639 Grenzach-Wyhlen, Germany; 9 F. Hoffmann-La Roche Ltd., Grenzacherstrasse 124, 4070 Basel, Switzerland

**Keywords:** real-world evidence, real-world data, health technology assessment, health policies, guidelines, appraisal

## Abstract

Background: Real-world evidence (RWE) can reinforce clinical trial evidence in health technology assessment (HTA). Objectives: Review HTA bodies’ (HTAbs) requirements for RWE, real uses, and acceptance across seven countries (Brazil, Canada, France, Germany, Italy, Spain, and the United Kingdom) and outline recommendations that may improve acceptance of RWE in efficacy/effectiveness assessments and appraisals processes. Methods: RWE requirements were summarized based on HTAbs’ guidelines. Acceptance by HTAbs was evaluated based on industry experience and case studies. Results: As of June 2022, RWE methodological guidelines were in place in three of the seven countries. HTAbs typically requested analyses based on local data sources, but the preferred study design and data sources differed. HTAbs had individual submission, assessment, and appraisal processes; some allowed early meetings for the protocol and/or results validation, though few involved external experts or medical societies to provide input to assessment and appraisal. The extent of submission, assessment, and appraisal requirements did not necessarily reflect the degree of acceptance. Conclusion: All the countries reviewed face common challenges regarding the use of RWE. Our proposals address the need to facilitate collaboration and communication with industry and regulatory agencies and the need for specific guidelines describing RWE design and criteria of acceptance throughout the assessment and appraisal processes.

## 1. Introduction

Regulatory agencies are responsible for ensuring that the benefits of medicines outweigh their risks. Randomized controlled trials (RCTs) are the gold standard for demonstrating efficacy (Table 1) and safety in accordance with the principles of evidence-based medicine [1]. RCTs thus provide data on the relative effects of a new treatment, albeit in very controlled environments, which may limit the generalizability (Table 1) of the results. The highly specific framework of an RCT, including the stringent monitoring of treatment administration, the defined follow-up time, and the strict selection criteria, ensures that the study remains internally valid. However, these experimental conditions may not reflect real-life practice, for example, if the RCT has excluded certain patient populations or if it was conducted over a relatively short time horizon (e.g., 1–2 years) [2]. As a result, a data gap arises between efficacy (as assessed in RCTs) and real-world effectiveness (Table 1) as emulated from real-world data (RWD, Table 1), which refers to drug responses assessed in routine clinical practice. Whilst regulatory agencies use RCT-generated evidence to assess the efficacy and safety of a new medicine, health technology assessment (HTA) bodies and healthcare payers need to understand the cost-effectiveness and value for money a new therapy may bring to patients and the healthcare system [3,4,5]. This highlights the need to complement RCT data with RWD [6] to close the efficacy–effectiveness data gaps [7,8,9,10,11].

Real-world evidence (RWE, Table 1) generated from hypothesis-evaluating treatment effectiveness studies (RWD-HETE) can be used to narrow the efficacy–effectiveness gap. These particular types of studies (as opposed to exploratory RWD [12]) allow for an effectiveness assessment (Table 1) based on a given hypothesis related to a real-life setting (e.g., considering real-life compliance of the treatment under study or focusing on populations not represented in the RCTs). RWE also represents meaningful information when direct comparisons are not feasible within an RCT, such as when the new treatment under study is unique and lifesaving and including a control arm would be unethical.

Additionally, recent advances in personalized medicine (e.g., drugs that can target specific mutations often occurring in very small-sized populations) support the use of real-world post-authorization studies. Some noted examples of instances where decision makers have leveraged RWE include the post-market studies for long-term effectiveness and safety follow-up, as well as the assessment of health-related quality of life or healthcare resources consumption [2,9,12,13,14,15,16].

How HTA bodies consider RWD and RWE in decision making—i.e., their acceptance (Table 1)—is, therefore, an increasing topic of importance for the assessment of health products [15,16,17,18]. Methodological aspects, such as the robustness of real-life studies and the scientific validity of their results, are crucial for consideration in the evaluation of health products [2,16,19,20,21,22].

This analysis aims to understand HTA bodies’ stated requirements for RWE, as well as real uses and acceptance across seven countries (Brazil, Canada, France, Germany, Italy, Spain, and the United Kingdom) based on experiences from industry and to outline recommendations that may drive acceptance of this complementary evidence.

## 2. Materials and Methods

### 2.1. Sources of Data and Scope of the Survey

Data on the acceptance of RWE by HTA bodies were analyzed according to the experiences of Roche, a pharmaceutical company that has conducted many HTA submissions globally and has regularly included RWE in these submissions. Data from seven countries (Brazil, Canada, France, Germany, Italy, Spain, and the United Kingdom, representing the largest ex-US pharma markets with established HTA systems in place) were collected through an internal questionnaire that covered the following: (1) methodological requirements based on HTA bodies’ guidelines, when available; (2) real uses of RWE in HTA submissions based on Roche’s subsidiaries experience in each country; and (3) RWE acceptance based on case studies (Table 2).

### 2.2. Conception of the Questionnaire

The questionnaire was conceived through the collaboration of Roche employees and external consultants (IQVIA). This resulted in the definition of the main topics and outcomes (both qualitative and quantitative) to be included in the questionnaire, assessed by multiple choice and closed-ended questions to ensure reproducibility. The selected topics were as follows:

- Methodological requirements section (21 questions): guidelines, outcomes, type of studies, methods, and data sources;
- Real uses section (16 questions): submission of RWE in the HTA process, submitted data, submission process, assessment, and advocacy;
- Acceptance (case studies) section (23 questions): product/indication identification, context of RWE submission, RWE submitted, outcomes, and acceptance.

### 2.3. Completion of the Questionnaire

The questionnaire was implemented as a Google form to facilitate data collection, extraction, and analysis. The questionnaire was completed by team members from all concerned Roche subsidiaries (hereafter named the respondents) between March and June 2022. In completing the questionnaire, each Roche subsidiary involved one or more contributors based on their expertise; regardless of the number of people involved, all the contributions were synthesized in one form for each subsidiary. Each questionnaire could either be completed online or discussed orally if respondents needed support to finalize and/or validate answers. The methodological requirements section was completed based on official guidelines from HTA bodies, where available (Table 2). Real uses were identified based on the respondents’ experience. The case studies questionnaire section was filled based on experience with RWE data submission by each respondent. Whenever possible, respondents presented three examples of RWE usage: one base case (RWE “partially accepted”, where RWE is positively mentioned in the HTA assessment and considered as valid to inform but its content does not impact decision making on access and reimbursement (Table 1)), one best case (RWE “accepted”, where RWE is taken into consideration to impact decision making on access and reimbursement) and one worst case (RWE “not accepted”, where it is mentioned in the HTA assessment that RWE was not considered for appraisal (Table 1), or not mentioned at all). Results have been extracted and gathered to obtain general results for each topic.

## 3. Results

### 3.1. Local Methodological Guidance and Templates

At the time of completing the questionnaires (June 2022), four out of the seven countries’ HTA bodies included in this study (Brazil, Canada, Italy, and Spain) did not have formally published methodological guidance on RWD and RWE use (Table 3 and Table S1). Only France (HAS, French Health Authority), the United Kingdom (UK, NICE, National Institute for Health and Care Excellence), and Germany (IQWiG, Institute for Quality and Efficiency in Healthcare) had published methodological guidelines on RWD and RWE usage during the same period (Table 3, Table 4 and Table S1) [2,23,24].

In France and the UK, the methodological guidance (Table 4) [2,23] was considered sufficiently clear and detailed regarding how RWE can be used in benefit assessments (Table 3 and Table S2). In fact, the guidelines contained information about how to assess outcomes, structure study designs, select data sources, define protocol requirements, identify the study population, and minimize bias. In addition, both bodies guided the usage of RWE in external control arms [2,23,28]. Interestingly, only France had templates (Table 1) for drafting the study protocol and the study report (Table 3 and Table S1). These templates were considered useful by the respondents in fulfilling methodological requirements (i.e., study population, external arms, bias minimization).

### 3.2. Guidance for RWE Acceptance from HTA Bodies

Regarding RWE acceptance from HTA bodies, none of the seven countries had published specific guidance aimed at specifying when RWE use is accepted (Table 3).

### 3.3. Type of Outcomes and Sources of RWD Submitted

In practice, a wide variety of RWD study designs could be submitted within an HTA dossier, depending on the scope of the assessment (e.g., clinical benefit assessment, reimbursement assessment decision, health–economic assessment, and price setting) (Table 3 and Table S2). According to the respondents, the HTA bodies’ preferred type of outcomes regarding efficacy/effectiveness assessment focused mainly on the “efficacy gap between RCT and RWD”, “comparative effectiveness”, and “relevance of medicine’s effect”, followed by “quality-of-life assessment” and “long-term analyses” (Table S2). RWD could be derived from multiple sources. Healthcare databases, including electronic health records (EHRs) and patient registries, were the most widely used (Table 3 and Table S2). Responses to the questionnaire suggest that HTA bodies preferred local RWD sources to put results into perspective in clinical care within the country and ensure their transposability to the eligible population (Table S2).

HTA bodies from Germany, France, and Brazil had identified a list of existing relevant RWD sources to inform and impact decision making on access and reimbursement, but this was absent in Canada, Italy, Spain, and the UK (Table 3 and Table S1).

### 3.4. External Expertise for RWE-Based HTAs

Most countries did not involve an external review group (ERG) in the protocol and results validation. Only the UK, Germany (IQWiG is considered as an ERG acting on behalf of the German Federal Joint Committee G-BA), and Spain included ERGs in study assessment and appraisal. The UK was the only country known to consider ERG conclusions in its final appraisal (Table 3 and Table S2).

The answers to the questionnaire suggest that all countries involved scientific academics, experts, or key opinion leaders (KOLs) in the different aspects of RWE assessment and appraisal. Their main roles referred to publications on RWD use, advocacy on the use of RWD (Brazil, France, and Italy), and sometimes consultation in assessment and appraisal steps (Brazil, Canada, Germany, and the UK) (Table 3 and Table S2).

### 3.5. Feasibility of Early Consultations around RWE-Based HTA Dossiers

In most countries (Brazil, Canada, France, Germany, and the UK), early consultations with the HTA body were authorized, but they were not dedicated specifically to RWD (Table S2). On the other hand, an application for an early meeting with an HTA body was found not to be possible in Spain and hardly feasible in Italy.

### 3.6. Acceptance of RWE from 12 Case Studies

Case studies were used to understand the relationship between a study protocol and acceptance of RWE by HTA bodies. Seven countries of our analysis reported 12 case studies. In four cases, the HTA body evaluated the protocol (Brazil, Canada, Italy, and the UK) and considered outcomes based on RWD during appraisal. For those four studies, RWD were accepted (*n* = 3) or partially accepted (*n* = 1) from the applicant’s point of view. For the eight other case studies, without a protocol evaluation by the HTA body, RWD were either partially accepted (*n* = 3) or not accepted (*n* = 5) during appraisal (Table 5 and Table S3).

## 4. Discussion

The aim of the present analysis was to understand HTA bodies’ stated requirements for acceptance of comparative RWE across seven countries (Brazil, Canada, France, Germany, Italy, Spain, and the United Kingdom) and outline recommendations that may improve acceptance of this evidence within drug appraisal processes by HTA bodies.

The relevance and use of RWD to generate RWE for a medicine’s assessment and appraisal has grown considerably with recent advances in data collection and analytics [13,29,30]. RWE can provide additional information on the clinical benefit of medicines in real-life practice settings, thus representing a powerful complement to overcome the inherent limitations of pivotal RCTs, such as population generalizability and transportability.

From this case study evaluating RWE guidelines and acceptance of non-randomized comparative studies, five key findings of the present analysis lead to recommendations on how to expand the use of RWD/RWE in drug appraisal processes by HTA bodies.

### 4.1. Most of the Studied HTA Bodies Do Not Have Methodological Guidance or Templates

Guidance and/or a framework for RWE acceptance is necessary to clearly identify HTA bodies’ expectations regarding the use of RWD so as to ensure that all criteria of relevance leading to health decisions are followed [31]. Different frameworks have been published, such as the real-world evidence program of the US FDA describing conditions of RWD use, adequate study design, regulatory requirements [32], and more recently, RWD use in external control arms to single-arm clinical trials, the WHO-INTEGRATE evidence-to-decision framework based on the norms and values of the WHO [31,33], and the European OPeratIonal, TechnIcal, and MethodologIcAL (OPTIMAL) framework which aims to define the appropriate use of valid RWE for regulatory purposes [34]. Furthermore, the Canadian Real-world Evidence for Value of Cancer Drugs (CanREValue) collaboration, involving researchers, recommendation-makers, decision makers, payers, patients, and caregivers, is also developing a framework for the reassessment of cancer drugs in the real-life setting [35,36].

There is, however, a need to develop methodological guidelines regarding possible RWE usage, including best practices to improve current standards. At the data cut-off date of the present analysis (June 2022), only France, Germany, and the UK had methodological guidance regarding RWE use to inform decision making, and only France had developed a specific template for RWE reporting. Among the methodological guidance, the UK had the most advanced RWE framework to support companies in the generation of RWE. As a result, in the UK, RWD were used in almost all the NICE reports on cancer treatments between 2011 and 2016 [15,16]. In contrast, only 37.7% of initial marketing authorization applications on antineoplastic and immunomodulating agents submitted at the European Medicines Agency (EMA) between 2018 and 2019 were based on RWD [14].

In Italy, the Italian Pharmacology Society (SIF) has taken a step in this direction, acknowledging the integrative role of RWE versus RCTs, particularly in the regulatory and health planning spheres. Afterwards, a reference document for RWD/RWE considerations was issued by ISPOR Italy in late 2022 [26]. However, the endorsement and enhancement of such an expert position by the Italian Medicine Agency (AIFA) is a prerequisite for the concrete implementation of RWE in HTA processes. Hence, the country still lacks official guidelines issued by AIFA on how to collect, analyze, and interpret RWD to reinforce evidence from RCTs for HTA purposes, which is particularly useful when the latter alone is not sufficient to make informed decisions on drugs (e.g., single-arm trials).

Thus, all countries should advocate for referenced templates and methodological guidance, including RWD methodologies that provide supporting evidence for new types of clinical trials [37], particularly single-arm trials and the use of external comparators. This would direct pharmaceutical companies’ investment in RWD-based evidence generation by aligning it with the needs of HTA bodies and health systems, contributing to faster patient access to new therapies. Moreover, the existence of guidelines including templates—e.g., HARPER protocol template [38]—would facilitate the generation and use of high-quality RWE [19] and, in turn, support HTA bodies’ confidence in RWD sources and methods.

Our results suggest that even though guidelines were soundly used as methodological support, they had a limited impact on HTA decision making so far. How HTA bodies leverage RWE in their decision-making processes thus remains uncertain. The NICE framework goes further on this issue by describing the circumstances of relevant uses of RWD in HTA, i.e., no relevant control arm, limited population follow-up, and generalizability [23], reflecting Roche’s position [39].

Specific guidance describing the HTA body criteria of acceptance of RWE would ensure the use of RWE for drug appraisal is appropriately generated. This guidance could include a list of typical use cases where HTA bodies would expect (and accept) RWE. This would increase the efficiency of data-driven decision making.

### 4.2. Most of the Studied Countries Can Submit a Variety of Study Results Leveraging Different RWD Sources, Depending on the Scope and Objective of the Assessment

In our analysis focusing on effectiveness, HTA bodies are familiar with answering a wide variety of objectives, including the gap between RCT and RWD in comparative efficacy/effectiveness and in the relevance of product effect. In these cases, EHR databases and patient registries were most frequently used. In most studied countries, an official list of RWD sources (library of databases) is missing [40], and when available, it includes mainly non-exhaustive data sources without a clear view of how the datasets were assessed to determine their quality or whether they are fit for use in the context of drug evaluation or appraisal. Among the assessed countries, only Germany (IQWiG) had dedicated and clear guidelines for RWD sources [41]. On the other hand, in Brazil and France, such listings were neither exhaustive nor detailed for each therapeutic area. Brazil recommended sources only for health–economic assessment (DATASUS [42], BPS [43], and CMED [30]). France currently has only a non-exhaustive data sources index; nevertheless, the latter could quickly evolve as the HAS has recently launched a call for the production of an inventory of all available RWD data to meet future requests for drug appraisals [29]. As of 9 November 2022, only six databases and real-life studies submitted as part of this call are “likely to be used to respond to requests for additional data from the French health authorities (HAS) for the evaluation of products and technologies”, all with the intention of use in post-registration studies [29]. In Italy, data from compassionate-use programs can be used in support of RCT data since 2017 (Ministerial Decree 7 September 2017 [44]), while the law foresees that observational studies as well as compassionate use registries can generate evidence for marketing authorization purposes [45]. In addition, the expert position on RWD/RWE issued by ISPOR in 2022 provides a comprehensive list of potential RWD sources that can be associated with different HTA purposes [26]. Nevertheless, neither these sources nor others have yet been formalized in a structured list of RWD sources by AIFA. Similarly, in the other countries, no RWD source list referenced by the HTA bodies as valuable for HTA submissions was available at the time of this analysis.

Regarding European marketing authorization, the main objectives of RWE studies included in applications between 2018 and 2019 were related to safety (87.3%) and effectiveness (49.2%). The most common sources of RWD were registries (60.3%) and hospital data (31.7%) [14]. An analysis of the use of RWD in HTA of melanoma drugs by five European HTA bodies between 2011 and 2016 reported that RWD were included in 54% of relative effectiveness assessments, mainly on epidemiologic considerations, and in 88% of cost-effectiveness assessments, mainly to estimate long-term effectiveness and/or costs [16]. In another analysis on cancer drugs for single technology appraisals, conducted by NICE between 2011 and 2018, RWD were included mainly to assess quality of life (71%), cost (46%), and medical resources utilization (40%) [15]. Additionally, RWD are increasingly being used to serve as external control arms of single-arm trials [13].

RWD sources and methodology need to be further improved in several countries to improve RWE acceptance in practice [16,17,22,46,47]. There is a need to standardize RWD to improve the data processing by the decision makers [9,15], as was performed for the clinical trials within the Clinical Data Interchange Standards Consortium (CDISC) [48] and the Observational Medical Outcomes Partnership (OMOP) Common Data Model (CDM) [49]. The joint ISPOR-ISPE special task force on real-world evidence in healthcare decision making published recommendations for RWD from hypothesis-evaluating treatment effectiveness (HETE) studies [12,50]. The DARWIN EU project [51] and, more globally, the European Big Data project [52,53] aim to establish a catalog of observational data sources for use in medicines regulation and provide a source of high-quality, validated RWD on the uses, safety, and effectiveness of medicines. In its real-world evidence program, the US FDA aims to define regulatory requirements regarding the use of RWD and to develop data standards [32].

Patient registries are also important sources of RWD, and the European network for Health Technology Assessment (EUnetHTA) [22] promotes their use in HTA. For the German IQWiG, high-quality registries are more suitable for post-launch RWE to assess the benefit of a new drug in comparison to other RWD, such as electronic health records and claims databases, which are considered less reliable [48].

Local data which reflect the local population and standard of care are preferable [17,32,48]. Even though the UK’s NICE has a high level of RWD acceptance, the Evidence Review Group and decision makers can potentially reject RWD sources that do not represent the UK population [48]. Local reimbursement and pricing decisions must be based on data (clinical trial data and RWD) that reflect the local population and healthcare settings [17].

Therefore, manufacturers should ensure that the RWD source used is appropriate and relevant to answering the research questions at hand. Meanwhile, HTA bodies could provide more transparency around how RWD data sources are appraised and evaluated for quality and suitability to the research question. This could be achieved by (1) homogeneity and transparency regarding quality measures of a data source; (2) developing a framework to ensure the quality, methodological robustness, and usability of RWD for regulatory and HTAs; (3) establishing audit trails of RWD data sources; (4) listing previously acceptable data sources; and (5) improving access to recommended data sources here for HTA bodies around data sources.

### 4.3. Most HTA Bodies Do Not Involve an External Review Group for Assessment and Appraisal

Involvement of ERGs for an RWD protocol and results validation is not consistent among HTA bodies. Nevertheless, within all of the countries, scientific academics, experts, or KOLs are involved in the different aspects of RWE assessment and appraisal, either developing publications on RWD use, advocating the use of RWD (Brazil, France, and Italy), or sometimes consulting in assessment and appraisal steps. Only the UK includes recommendations or conclusions from ERGs in final appraisals [15]. This practice should be encouraged by other HTA bodies. The US FDA, as a part of its RWE program, proposes a review of the RWD/RWE-driven work by external stakeholders (industry, academics, patient advocacy groups) to identify specific needs to be addressed and to facilitate the use of RWE in regulatory decision making [32]. In the European DARWIN EU project, a dedicated advisory board, including EMA members, representatives of the European Union and the national competent authorities, payers, HTA representatives, patient associations, and healthcare professionals will be responsible for providing strategic advice on the usage of RWE and recommendations [51].

HTA bodies could provide RWE expertise for local drug appraisals and develop verification and sensitivity analyses based on submitted RWD and/or other sources of RWD. They could also include an independent methodological group in charge of the validation of the protocol according to HTA expectations and results using the official local methodological and RWD acceptance guidance. External review groups (scientific academics, therapeutic area experts, patient representatives, and key opinion leaders) could be involved at every step of the RWE assessment and appraisal process.

### 4.4. Some HTA Bodies Do Not Have an Early Consultation Process

Early scientific meetings with an HTA body provide an opportunity to discuss RWD generation, including the conception of the study and the relevance of the evidence being generated, which can improve its acceptance.

In light of the present results, it is likely that some HTA bodies may not have enough resources in RWD/RWE issues to organize sessions specifically dedicated to RWE, to implement these sessions on a regular basis, and to provide scientific advice regarding the implementation of RWE for decision making.

However, consultation meetings between the health authorities and companies are needed before initiating an RWE project [54]. To optimize those consultations, guidance on RWE acceptance by HTA bodies is necessary.

Early meetings based on a clear, dedicated process with HTA bodies are recommended before the RWE generation plan. They should involve independent external review groups as well as RWD providers.

In Europe, the new regulation on HTA (HTAR) [55] paves the way to increase early scientific dialogue and the consequent early planning of evidence generation in support of launch and/or post-launch HTA decision making. In fact, based on the HTAR, a joint scientific consultation (JSC) will issue scientific advice clarifying the evidence gaps that medicine developers should fill with a view to both the EU regulatory assessment (joint clinical assessment—JCA) [55] and the negotiation with national HTAbs. In the case of marketing authorizations, early JSC consultations could address the pivotal study design and the definition of evidence-generation plans supporting JCA and national reimbursement procedures, ultimately easing patient access. On the other hand, in the case of renegotiations, they could guide post-license evidence generation, thus facilitating the process. However, for this system to work properly, a joint effort to adopt and adapt to the regulation is needed by public and private stakeholders at both national and European levels.

### 4.5. There Is a Lack of Specific Guidance Aimed at Specifying When RWE Use Is Accepted within Drug Appraisal and for Which Objective

Detailed guidelines on RWD and RWE criteria of acceptance within HTA decisions and transparency of RWE consideration and impact within HTA assessment and appraisals will likely consolidate RWE submissions and could accelerate the availability of innovative medicines to patients in need.

## 5. Conclusions

In conclusion, while disparities exist between global HTA agencies on the acceptance of RWE, countries seem to face common challenges to enhance the potential of RWD. There is a need for HTA bodies and health authorities to define and optimize the generation of high-quality RWD accepted for drug appraisal and to facilitate their collaboration and communication with pharmaceutical companies and external stakeholders around complementary RWD packages for HTA.

## Figures and Tables

**Table 1:** Glossary.

Term	Definition
Acceptance	An HTA body considers the content of a real-world evidence study as having an impact on decision making for access and reimbursement
Appraisal	Valuation of the assessment results that support decision making
Assessment	Technical and scientific assessment of the data package
Efficacy	Ability of a medical product/indication to achieve a specific outcome in a clinical trial
Effectiveness	Ability of a medical product/indication to achieve a specific outcome in a real-life setting
Generalizability	The extent to which the findings of a clinical trial can be applied to a real-life setting
Partial acceptance	An HTA body considers a real-world evidence study as valid to inform, but its content does not impact decision making on access and reimbursement
Real-world data	Data relating to patient health status and/or the delivery of healthcare routinely collected from a variety of sources collected outside a clinical trial in the everyday clinical practice
Real-world evidence	Derived from the analysis of real-world data, it is the clinical evidence about the usage and potential benefits or risks of a medical product
Template	A structured report outline provided by an HTA body with defined sections

**Table 2:** HTA bodies per country.

Country	HTA Body
Brazil (BR)	Comissão Nacional de Incorporação de Tecnologias no Sistema Único de Saúde (Conitec) Drug Market Regulation Chamber (Camara de Regulaçao do Mercado de Medicamentos, CMED) *
Canada (CA)	Canada’s Drug and Health Technology Agency (CADTH) National Institute of Excellence and Social Services (Institut National d’Excellence en Santé et en Services Médicaux, INESSS)
France (FR)	National Health Authority (HAS)
Germany (DE)	The Federal Joint Committee (Gemeinsamer Bundersausschuss, G-BA) The Institute for Quality and Efficiency in Healthcare (Institut für Qualität und Wirtschaftlichkeit im Gesundheitswesen, IQWiG): independent scientific institute; examines the benefits and harms of medical interventions for patients
Italy (IT)	Italian Medicines Agency (Agenzia Italiana del Farmaco, AIFA)
Spain (ES)	Health Minister of Health (Ministerio de Sanitad Servicios Sociales e Igualdad, MSSSI) Spanish Agency of Medicines and Medical Devices (Agencia española de Medicamentos y Productos Sanitarios, AEMPS)
United Kingdom (GB)	The National Institute for Health and Care Excellence (NICE)

* Not an HTA agency but was considered in the evaluation as CMED is responsible for pricing evaluation prior to the HTA submission process in the country.

**Table 3:** Overview of results (methodological requirements and real use).

Outcomes	Absence ^a^	Presence ^a^
**Methodological Requirements**		
Methodological guidance from HTA body ^b^	4	3
	BR, CA *, IT *, ES	FR, DE, GB
Template for RWD submission from HTA body	6	1
	BR, CA, DE, IT, SP, GB	FR
Guidance for RWD acceptance from HTA body	7	0
	BR, CA, FR, DE, IT, ES, GB	/
RWD sources’ index	4	3
	CA, IT, ES, GB	BR, FR, DE
**Real uses**		
Type of assessments	Wide variety	
	TOP 3 1—Efficacy gap between RCT and RWD	
	2—Comparative effectiveness	
	3—Relevance of product/indication effect	
Type of RWD sources ^c^	Wide variety	
	TOP 2 1—Healthcare databases, including HER	
	2—Patient’ registries	
Preference for local RWD sources ^b^	Majority	
Involvement of ERG from HTA body for RWD protocol and results validation	4	3
	BR, CA, FR, IT	DE, ES, GB
Involvement of learned societies, KOLs, or experts to recognize RWD	0	7
	/	BR, CA, FR, DE, IT, ES, GB
Publication on RWD use	2	5
	DE, IT	BR, CA, FR, ES, GB
Consultation in submission and appraisal steps	3	4
	FR, IT, ES	BR, CA, DE, GB
Advocating on the use of RWD	5	2
	CA, FR, DE, ES, GB	BR, IT

^a^ Total of respondents for which the answer is “Yes”. ^b^ At the time of the data collection (cut-off June 2022), methodological guidance was issued from HTA bodies in France [2], Germany [24], and the UK [23]; none were issued in Brazil, Canada, Italy, and Spain; * The Canadian HTA body issued draft guidelines in November 2022 [25]. In Italy, there are still no official guidelines on RWE issued by the Italian Medicines Agency; reference documents are a report from ISPOR Italy [26] and an Expert position publication of the Italian Pharmacology Society (SIF) [27]. ^c^ For these items, questions were assessed according to the HTA steps, which is why results are reported in a qualitative way for the complete HTA process. Abbreviations: BR, Brazil; CA, Canada; FR, France; DE, Germany; HTA, health technology assessment; IT, Italy; KOL, key opinion leader; RCT, randomized clinical trial; RWD, real-world data; RWE, real-world evidence; SP, Spain; UK, United Kingdom.

**Table 4:** Local methodological guidance for RWD acceptance per country.

Countries	HTA Body Involved	Name of the Methodological Guideline	Ref.
France (FR)	HAS (French Health Authority)	Methodological guide: real-world studies for the assessment of medicinal products and medical devices	[2]
Germany (DE)	G-BA (Gemeinsame Bundesausschuss), G-BA commissions IQWiG, Institute for Quality and Efficiency in Healthcare)	Concepts for the generation of routine practice data and their analysis for the benefit assessment of drugs according to §35a Social Book V	[24]
United Kingdom (GB)	NICE (National Institute for Health and Care Excellence)	NICE Real-world Evidence Framework	[23]

**Table 5:** List of case studies with final HTA decision to consider/not consider RWD/RWE submission.

Name	DCI	Country	Agency	Indication	Year of Appraisal	Therapeutic Area	Outcomes	Sources	Level of RWE Acceptance
POLIVY	Polatuzumab	BR	CMED *	Relapsed or refractory diffuse large B-cell lymphoma (2L+)	2021	Oncohematology	Comparative efficacy vs. any clinically relevant comparator	Healthcare databases, including EHRs	Partially accepted
EVRYSDI	Risdiplam	BR	CONITEC	SMA type 1, 2, 3	2021	SMA	Microcosting	Patient registries	Accepted
ALECENSA	Alectinib	CA	CADTH	Anaplastic lymphoma kinase-positive, locally advanced (not amenable to curative therapy), or metastatic NSCLC who have progressed on or are intolerant to crizotinib until loss of clinical benefit	2018	Oncology	Comparative efficacy vs. any clinically relevant comparator	Healthcare databases, including EHRs	Not accepted
ROZLYTREK	Entrectinib	CA	CADTH	First-line treatment of patients with ROS1-positive locally advanced or metastatic NSCLC	2021	Oncology	Comparative efficacy vs. any clinically relevant comparator	Healthcare databases, including EHRs	Not accepted
POLIVY	Polatuzumab	CA	CADTH	In combination with bendamustine and rituximab for relapsed or refractory diffuse large B-cell lymphoma, not eligible for ASCT and have received at least one prior therapy	2021	Oncohematology	Difference between efficacy in RCT and RWD	Healthcare databases, including EHRs	Partially accepted
ALECENSA	Alectinib	DE	G-BA	Anaplasic lymphoma kinase (ALK) positive advanced NSCLC	2017	Oncology	Generation of a control arm	Healthcare databases, including EHRs	Not accepted
ROZLYTREK	Entrectinib	ES	MoH	NTRK and ROS1	2021	Oncology	Comparative efficacy vs. any clinically relevant comparator	Patient registries and healthcare databases, including EHRs	Not accepted
PERJETA	Pertuzumab	ES	MoH	Breast cancer HER2	2020	Oncology	Difference between efficacy in RCT and RWD	Healthcare databases, including EHRs	Partially accepted
ROZLYTREK	Entrectinib	FR	HAS	Advanced forms of ROS1 + NSCLC, not previously treated with ROS1 inhibitors	2021	Oncology	Comparative efficacy vs. any clinically relevant comparator	Patient registries, ESME cohort	Not accepted
TECENTRIQ	Atezolizumab	FR	HAS	Advanced or unresectable HCC who have not received previous systemic therapy	2021	Oncology	Difference between efficacy in RCT and RWD	Patient registries, compassionate use program	Partially accepted
TECENTRIQ	Atezolizumab	GB	NICE	Second-line metastatic NSCLC	2017	Oncology	Difference between efficacy in RCT and RWD	Patient registries	Accepted
ESBRIET	Pirfenidone	IT	AIFA	Pulmonary fibrosis	2021	Pneumology	Long-term efficacy to remove a treatment-stopping rule	Healthcare databases, including EHRs	Accepted

* Not an HTA agency but was considered in the evaluation as CMED is responsible for pricing evaluation prior to the HTA submission process in the country. Abbreviations: 2L+, second line of treatment; ASCT, autologous stem cell transplant; CADTH, Canadian Agency for Drugs and Technologies in Health; CMED, Câmara de Regulação do Mercado de Medicamento; CONITEC, Comissão Nacional de Incorporação de Tecnologias no SUS; EHRs, electronic health records; ESME, Épidémio-Stratégie Médico-Économique; G-BA, the German federal joint committee (Gemeinsamer Bundersausschuss); HAS, Haute Autorité de Santé; HCC, hepatocellular carcinoma; HER2, human epidermal growth factor receptor 2; MoH, Ministry of Health; NICE, National Institute for Health and Care Excellence; NTRK, neurotrophic tyrosine receptor kinase; NSCLC, non-small-cell lung cancer; RCT, randomized controlled trial; ROS1, ROS protooncogene 1; RWD, real-world data; SMA, spinal muscular atrophy.

## Data Availability

The original contributions presented in this study are included in the article/supplementary material; further inquiries can be directed to the corresponding author.

## Supplementary Materials

The following supporting information can be downloaded at https://www.mdpi.com/article/10.3390/jmahp12020009/s1, Table S1: Main results of the methodological requirements questionnaire section; Table S2: Main results of the real uses of RWD and RWE questionnaire section; Table S3: Main results on acceptance from a list of case studies.
