# Supplementary material for: Real-World Evidence to Reinforce Clinical Trial Evidence in Health Technology Assessment: A Critical Review of Real-World Evidence Requirements from Seven Countries and Recommendations to Improve Acceptance
Source: J Mark Access Health Policy. 2024 May 20;12(2):105–17. doi: 10.3390/jmahp12020009 (PMC11130860; doi:10.3390/jmahp12020009)
Supplement: Supplementary file 1 [file jmahp-12-00009-s001.zip › jmahp-2868134-supplementary.pdf]

Journal of
Market Access & Health Policy

MDPI

Supplementary materials

Table S1: Main results on the Methodological requirements questionnaire section

Outcomes	Total[a]	Respondents[b]	Brazil	Canada	France	Germany	Italy	Spain	United-Kingdom
**Existence of templates for protocol & report**	1	7			X				
**Existence of official methodological guidance:**	3	7			X	X			X
That is considered as methodological support only	3	3			X	X			X
**Information on outcomes and studies**[c]									
Type of outcomes that can be submitted according to methodological guidance:									
Effectiveness	3	3			X	X			X
Quality of life	2	3			X	X			
Cost-effectiveness	1	3							X
Type of studies that can be submitted according to methodological guidance:									
Comparative studies	3	3			X[d]	X			X
Descriptive studies	2	3			X[d]	X			
Cohort studies	2	3			X[d]				X
Registry-based studies	1	3				X			
Longitudinal studies	1	3			X[d]				
**Information on RWD sources**[c]<br>Data sources can be:									
Public	3	3			X	X			X
Private	3	3			X	X			X
Existence of RWD sources' indexes	3	3	X		X	X			
Recommendations on transparency measures for data sources	2	3			X	X			
**Information on protocol & study report's requirements**[c]<br>Methodological guidance provide information on:									
Population requirements	3	3			X	X			X
External arm requirements	2	3			X				X

Bias minimization	3	3	X	X	X

a Total of respondents for which answer is “Yes”, b Number of respondents for each question, c Only countries that have official methodological guidance (n=3) answer to these items, d In France, types of studies depend on type of outcomes submitted. Cohort and comparative studies are preferred for effectiveness outcomes. Longitudinal comparative or descriptive studies are preferred for quality-of-life outcomes. X: Answer is "Yes”. Abbreviations: ERG, External review group; RWD, Real world data.

Table S2: Main results of the Real uses of RWD and RWE questionnaire section

Outcomes	Total[a]	Respondents[b]	Brazil	Canada	France	Germany	Italy	Spain	UK
INFORMATION ON SUBMISSION									
Early meetings with HTA body can be organized[c]	5	7	X	X	X	X			X
CLINICAL BENEFIT ASSESSMENT*									
HTA bodies involved			CMED/ CONITEC	CADTH / INESSS	HAS	G-BA / IQWiG	AIFA	MSSSI	NICE
RWD are:									
Mandatory	0	7							
Useful	6	7	X	X	X		X	X	X
Not accepted	1	7				X			
Type of outcomes that can be submitted in practice[d]:									
Efficacy gap between RCT and RWD	6	6	X	X	X		X	X	X
Comparative effectiveness	5	6	X	X	X			X	X
Relevance of product/indication effect	5	6	X	X	X		X		X
Quality-of-life outcomes	5	6	X	X	X		X		X
Estimate of comparative treatment effect	4	6	X	X			X		X
Long term quality of life outcomes	3	6	X	X					X
Long term effectiveness outcomes	3	6	X	X					X
Healthcare resources use	1	6							X
Costs	1	6							X
Utilities	0	6							
Local RWD sources[d]:									
Mandatory	1	6			X				
Useful	3	6		X			X		X
Neutral impact	2	6	X					X	
Type of RWD sources[d]:									

Healthcare databases including EHR	6	6	X	X	X		X	X	X
Patients' registries	5	6		X	X		X	X	X
Data supported by medical society	4	6		X	X		X	X	
Health insurance databases	3	6		X	X			X	
Pharmacy databases	3	6					X	X	X
Patient-powered research networks	3	6		X			X	X	
Social media	1	6						X	
REIMBURSEMENT ASSESSMENT DECISION									
HTA bodies involved			CONITEC	CADTH / INESSS	HAS - CT	-	AIFA - CTS	MSSSI	NICE
RWD are:									
Mandatory	0	7							
Useful	6	7	X	X	X		X	X	X
Not accepted	1	7				X			
Type of outcomes that can be submitted in practice[d]:									
Quality-of-life outcomes	6	6	X	X	X		X	X	X
Efficacy gap between RCT and RWD	6	6	X	X	X		X	X	X
Comparative effectiveness	5	6	X	X	X			X	X
Relevance of product/indication effect	5	6	X	X	X			X	X
Long term quality of life outcomes	5	6	X	X			X	X	X
Estimate of comparative treatment effect	4	6	X	X				X	X
Long term effectiveness outcomes	5	6	X	X			X	X	X
Healthcare resources use	3	6		X			X		X
Costs	6	6		X			X		X
Utilities	2	6		X			X		
Local RWD sources[d]:									
Mandatory	1	6			X				
Useful	4	6	X	X			X		X
Neutral impact	1	6						X	
Type of RWD sources[d]:									
Healthcare databases including EHR	6	6	X	X	X		X	X	X

Patients' registries	6	6	X	X	X		X	X	X
Data supported by medical society	5	6	X	X	X		X	X	
Patient-powered research networks	4	6	X	X			X	X	
Health insurance databases	3	6		X	X			X	
Pharmacy databases	3	6		X			X	X	
Social media	1	6						X	
HEALTH-ECONOMIC ASSESSMENT									
HTA bodies involved			CONITEC	CADTH / INESSS	HAS - CEESP	-	AIFA - CPR	-	NICE
RWD are:									
Mandatory	0	7							
Useful	5	7	X	X	X		X		X
Not accepted	2	7				X		X	
Type of outcomes that can be submitted in practice[d]:									
Quality-of-life outcomes	5	5	X	X	X		X		X
Long term quality of life outcomes	5	5	X	X			X		X
Efficacy gap between RCT and RWD	5	5	X	X	X		X		X
Comparative effectiveness	4	5	X	X	X				X
Relevance of product/indication effect	4	5	X	X	X				X
Estimate of comparative treatment effect	3	5	X	X					X
Long term effectiveness outcomes	4	5	X	X			X		X
Healthcare resources use	3	5		X			X		X
Costs	3	5		X			X		X
Utilities	2	5		X			X		
Local RWD sources[d]:									
Mandatory	3	5	X	X	X				
Useful	2	5					X		X
Neutral impact	0	5							
Type of RWD sources[d]:									
Healthcare databases including EHR	5	5	X	X	X		X		X
Patients' registries	5	5	X	X	X		X		X

Patient-powered research networks	2	5			X		X		
Data supported by medical society	2	5		X			X		
Health insurance databases	2	5		X	X				
Pharmacy databases	3	5		X	X		X		
Social media	1	5			X				
PRICE SETTING									
HTA bodies involved			CMED	pCPA	CEPS	GKV	AIFA - CPR	-	NICE
RWD are:									
Mandatory	0	7							
Useful	4	7		X	X		X		X
Not accepted	3	7	X			X		X	
Type of outcomes that can be submitted in practice[d]:									
Efficacy gap between RCT and RWD	4	4		X	X		X		X
Quality-of-life outcomes	3	4		X			X		X
Long term quality of life outcomes	3	4		X			X		X
Comparative effectiveness	2	4		X					X
Relevance of product/indication effect	2	4		X					X
Estimate of comparative treatment effect	2	4		X					X
Long term effectiveness outcomes	3	4		X			X		X
Healthcare resources use	3	4		X			X		X
Costs	3	4		X			X		X
Utilities	2	4		X			X		
Local RWD sources[d]:									
Mandatory	1	4			X				
Useful	3	4		X			X		X
Neutral impact	0	4							
Type of RWD sources[d]:									
Healthcare databases including EHR	4	4		X	X		X		X
Patients' registries	4	4		X	X		X		X
Data supported by medical society	3	4		X	X		X		
Patient-powered research networks	2	4		X			X		

Health insurance databases	2	4		X	X				
Pharmacy databases	3	4		X	X		X		
Social media	1	4							
IMPLICATION OF EXTERNAL EXPERTISE FOR RWE-BASED HTAS									
Information on protocol review & validation									
Implication of an external review group in protocol review & validation	3	7				X		X	X
Consideration of its conclusions in final appraisal[e]	1	7							X
Information on RWD recognition									
Implication of KOL, experts or learned societies	7	7	X	X	X	X	X	X	X
Roles:									
Publication on RWD's use	6	7	X	X	X		X	X	X
Consultation in submission & appraisal steps	5	7	X	X		X	X		X
Advocating on the use of RWD	3	7	X		X		X		

* Items answered by step of HTA process (only for information on submission items) : Clinical benefit assessment, Reimbursement assessment decision,
a Total of respondents for which answer is "Yes", b Number of respondents for each question, c Early meeting are accepted by HTA body and can be realized in practice, d Only countries that can submit RWD for the HTA step can answer to these items, e Only countries for which ERG is involved in protocol review answer this item, X: Answer is "Yes".
Abbreviations: AIFA-CPR, Italian Medicines Agency – Pricing and reimbursement committee; AIFA-CTS, Scientific Technical Committee; CADTH-INESSS, Canada's Drug and Health Technology Agency - Institute for Excellence in Health and Social Services; CEPS, Economic Committee for Medicinal Products, CMED, Drug market regulation chamber; CONITEC, National Commission for the Incorporation of Technologies; EHR, Electronic Health Record; GKV, German statutory health insurance; HAS, French health authority; HAS-CEESP, Commission for Economic and Public Health Evaluation; HAS-CT, Transparency committee; HTA, Health Technology Assessment; IQWIG, Institute for Quality and Efficiency in Health Care; KOL, Key Opinion Leader; MSSSI, Health minister of Spain; NICE, National Institute for Health and Care Excellence; pCPA, pan-Canadian Pharmaceutical Alliance; RCT, Randomized clinical trial; RWD, Real world data; UK, United Kingdom.

Table S3: Main results on acceptance from a list of case-studies

Outcomes	Total[a]	Brazil		Canada			France		Germany	Italy	Spain		United-Kingdom
Case-studies	12	1	2	3	4	5	6	7	8	9	10	11	12
Therapeutic area													
Oncology	10		X	X	X	X	X	X	X		X	X	X
Rare disease	1	X											
Pneumology	1									X			
HTA Body													
CONITEC	1	X											
CMED	1		X										
CADTH	3			X	X	X							
HAS – CT	2						X	X					
G-BA	1								X				
AIFA	1									X			
MSSSI	2										X	X	
NICE	1												X
Submission													
Type of outcomes													
Efficacy gap between RCT and RWD	4						X	X				X	X
Comparative effectiveness	5		X	X	X	X					X		
Costs	1	X											
External validity of RCT	1						X						
Generation of a control arm	1								X				
Other	1									X			
Data sources													
Patients' registry	6	X	X				X	X				X	X
Healthcare databases including EHR	6			X	X	X			X		X	X	
Cohort	2							X		X			
Compassionate use program	1						X						
Assessment													

Validation of protocol & data sources by HTA body	3	X		X						X			
Limitations highlighted	9	X	X	X	X	X	X	X	X	X			
Strengths highlighted	3	X		X						X			
Appraisal													
Protocol has been evaluated	4	X		X						X			X
Outcomes based on RWD were considered	4	X		X						X			X
Qualification of acceptance according to industrial													
Accepted	3	X								X			X
Partially accepted	4		X	X			X					X	
Not accepted	5				X	X		X	X		X		

Abbreviations: AIFA-CPR, Italian Medicines Agency; CADTH, Canada's Drug and Health Technology Agency; CMED, Drug market regulation chamber; CONITEC, National Commission for the Incorporation of Technologies; EHR, Electronic Health Record; HTA, Health Technology Assessment; G-BA, German federal joint committee; HAS-CT, Transparency committee; MSSSI, Health minister of Spain; NICE, National Institute for Health and Care Excellence; RCT, Randomized clinical trial; RWD, Real world data
